# Sequential aiming in pairs: the multiple levels of joint action

**DOI:** 10.1007/s00221-021-06060-5

**Published:** 2021-03-08

**Authors:** James W. Roberts, James Maiden, Gavin P. Lawrence

**Affiliations:** 1 Psychology, Action and Learning of Movement Laboratory (PALM), School of Health Sciences, Liverpool Hope University, Hope Park, Liverpool, L16 9JD UK; 2 School of Sport, Health and Exercise Sciences, Institute for the Psychology of Elite Performance, Bangor University, George Building, Bangor, LL57 2PZ UK; 3 Brain and Behaviour Laboratory, Research Institute of Sport and Exercise Sciences (RISES) Tom Reilly Building, Liverpool John Moores University, Byrom Street, Liverpool, L3 5AF UK

**Keywords:** Top–down, Bottom–up, Spatial variability, Feedforward, Action–observation

## Abstract

The task constraints imposed upon a co-actor can often influence our own actions. Likewise, the observation of somebody else’s movements can involuntarily contaminate the execution of our own movements. These joint action outcomes have rarely been considered in unison. The aim of the present study was to simultaneously examine the underlying processes contributing to joint action. We had pairs of participants work together to execute sequential aiming movements between two targets—the first person’s movement was contingent upon the anticipation of the second person’s movement (leader), while the second person’s movement was contingent upon the direct observation of the first person’s movement (follower). Participants executed separate blocks of two-target aiming movements under different contexts; that is, solely on their own using one (2T1L) and two (2T2L) of their upper limbs, or with another person (2T2P). The first movement segment generally indicated a more abrupt approach (shorter time after peak velocity, greater displacement and magnitude of peak velocity), which surprisingly coincided with lower spatial variability, for the 2T2P context. Meanwhile, the second segment indicated a similar kinematic profile as the first segment for the 2T2P context. The first movement of the leader appeared to accommodate the follower for their movement, while the second movement of the follower was primed by the observation of the leader’s movement. These findings collectively advocate two distinct levels of joint action including the anticipation (top–down) and mapping (bottom–up) of other people’s actions.

## Introduction

Joint action contexts often feature pairs of participants that undertake individual actions whilst working together. In these sorts of settings, individuals are typically influenced by a co-actor’s task constraints; in much the same way as if they themselves were faced with these constraints. This empirical observation has been mostly reflected within paradigms that have adapted a two-choice response task usually performed by one person (e.g., left- or right-sided response), and instead shared each of the possible responses between two persons (Sebanz et al. 2003; Tsai and Brass 2007; Tsai et al. 2008; Vlainic et al. 2010; Welsh et al. 2013; see also, Welsh et al. 2005). For example, it is well known that one of two possible cued responses (e.g., green-coloured symbol = left-sided response; red-coloured symbol = right-sided response) can become delayed when the cue is presented on the opposing side of space relative to the required response (e.g., green-coloured symbol appears on the right side) (classic Simon effect). Naturally, this delay in response no longer unfolds when individuals are alone and respond only to one cue instead of a possibility of two cues (e.g., respond to the green-coloured symbol; ignore the red-colour symbol). However, a delay when responding to only to one cue begins to emerge when two possible cued responses are divided between two persons that are paired together (e.g., green-coloured symbol = left-sided person; red-coloured symbol = right-sided person) (joint/social Simon effect). In this regard, responses to only one cue in pairs (i.e., one-choice task) can begin to resemble the responses to one of two possible cues in isolation (i.e., two-choice task). Consequently, it is suggested that individuals may represent a co-actor’s task constraints in a manner that is functionally equivalent to when responses are issued alone—something referred to as co-representation (cf. Dolk et al. 2011).

In a similar line of research, it has been shown that individuals’ movement characteristics may be influenced by the observation of a co-actor’s movements within real-time. This feature has been frequently demonstrated by the tendency for executed movements to become interfered with or adopt similar characteristics to simultaneously observed movements. For example, the requirement to execute a rapid discrete movement (e.g., index finger lift) in response to a numeric cue (e.g., “1”) can be substantially delayed when the irrelevant background stimulus also features a different (e.g., middle finger lift), as opposed to the same (e.g., index finger lift), category of movement (Brass et al. 2000; Liepelt et al. 2009; Press et al. 2007; for a meta-analysis, see Cracco et al. 2018). In a similar vein, the execution of a continuous straight-line arm movement (e.g., horizontal) can begin to deviate and fall more closely in line with the spatio-temporal characteristics of a simultaneously observed incongruent movement (e.g., vertical) (Kilner et al. 2003; Roberts et al. 2015; see also, Richardson et al. 2009; Schmidt et al. 1990). These findings have been predominantly attributed to the notion of an observed action priming a representation for the execution of that same action—something otherwise referred to as motor contagion (Blakemore and Frith 2005). In support of this conjecture, neurobiological techniques have highlighted a common neural network for observation and execution, where the mere observation of actions can activate the same neural regions that are responsible for the execution of those same actions (Fadiga et al. 1995; Hamilton and Grafton 2008; Iacoboni et al. 1999; Kilner et al. 2009; Molenberghs et al. 2009; Strafella and Paus 2000).

While the aforementioned lines of research similarly indicate the utilisation of a common representation for action, it is perhaps worthwhile reflecting on their differences to further highlight joint action processes. Namely, the notion of co-representation typically captures the discrete and sometimes blinded nature of joint action, where individuals may anticipate the movement of a co-actor prior to them even observing it. Meanwhile, the notion of motor contagion appears to more greatly comprise the continuous coupling between perception and action as they simultaneously unfold. Of interest, there have been recent attempts to more closely contrast these different settings, and what it may mean for our understanding of joint action. For example, pairs of participants were instructed to simultaneously execute discrete or continuous target-directed reaches with an obstacle in between each of the participants’ reaches (van der Wel and Fu 2015). In addition, the participants’ movements could be fully observed or occluded from view of their co-actor. The findings showed that the participants generated a higher trajectory when their co-actor had to move over an obstacle during discrete reaches regardless of whether they could observe the co-actor’s movement (see also, Griffiths and Tipper 2009). Therefore, the co-actor’s need to avoid the obstacle was appropriately captured without necessarily relying upon the coupling of observed and executed actions. However, a similarly higher trajectory was generated when the co-actor moved over an obstacle during continuous reaches, but only present when participants could simultaneously observe the co-actor’s movement. In this instance, there was not necessarily a concern surrounding the co-actor’s obstacle, but a greater influence of the observed action events. The authors adapted the minimal architecture perspective (Vesper et al. 2010) to explain their findings; that is, a top–down interpretation helps accommodate the prediction of others’ upcoming actions and the related task constraints, while a bottom–up coupling process underpins the monitoring of others’ actions as they unfold.

With this in mind, there have been comparatively limited attempts to incorporate the potential mediating influence of bottom–up and top–down factors within a single instance of joint action (for a similar argument, see Rocca and Cavallo 2018, 2020). Hence, there is perhaps some benefit to be served of having both the co-representation of the to-be-performed task (prospective influence within a leader of action), as well as the subsequent impact that observed movements may have on the response of the co-actor (retrospective influence within a follower of action). Consequently, the present study adapts a sequential aiming paradigm, where performers generate a rapid aiming movement toward one target before transitioning toward a second target that is further in the distance (e.g., Adam et al. 2000; Fischman and Reeve 1992). The joint action variant of this task involves executing the sequence in pairs with one person being designated as the *leader* who is responsible for initiating the sequence to the first target, while the other person represents the *follower* who continues the sequence toward the second target. Because this context features the use of separate upper limbs for each pair of participants, we additionally compared it to a context where participants would execute the same sequence alone, but with one limb aiming toward the first target, and then the other limb aiming toward the second target (e.g., initial right limb movement followed by an extension with the left limb; Khan et al. 2010; Lawrence et al. 2016; Reilly et al. 2017).

The role of the leader is synonymous with joint action contexts that are associated with the task co-representation framework (e.g., Sebanz et al. 2003) because any behavioural effects while occupying this role should manifest from the sharing of anticipated task constraints of the follower. Thus, it is predicted that the leader will adopt a more rapid approach to the first target to accommodate more time for the follower to reach for the second target. Moreover, the leader may attempt to constrain the trial-by-trial temporal variability to make the movement more predictable, and thus easier for the follower to prepare and coordinate their movement (Vesper et al. 2011; for a discussion on ‘coordination smoothers’, see Vesper et al. 2010). With this in mind, the temporal variability may positively co-vary with the degree of synchrony between the first and second segments (as indicated by pause times—time spent on the first target prior to the initiation of the second segment).

In terms of the follower, it is relevant to consider that this individual is additionally primed by the observed movement events of their partner prior to undertaking their own movement. Thus, we may attribute the hypothesized effects for this individual to the immediate or automatic coupling of perception and action (e.g., Heyes 2011; Hommel et al. 2001). As a result, it is predicted that the follower will undertake a similar spatio-temporal kinematic profile as the preceding co-actor including the displacement and time to/after peak velocity (e.g., Bisio et al. 2010; Wild et al. 2010; Hayes et al. 2014).

## Methods

### Participants

An a priori power analysis was conducted using G*Power software (version 3.1.9.4; see Faul et al. 2007) including the following input parameters: α = 0.05, 1 − β = 0.95, and *f* = 0.40 (large). There was a minimum requirement of 18 participants for this particular study. Twenty-six participants agreed to take part in the present study (age range = 18–40 years, 13 male, 13 female, 25 self-declared right-handed). Twelve pairs were gender-matched and one pair was mixed. There were two participants from different pairs that were removed following collection due to data recording errors across a large portion of their movement trials (remaining *n* = 24). The study was approved by the local Research Ethics Board, and designed and conducted in accordance to the Declaration of Helsinki (1964).

### Apparatus, task and procedure

**Fig. 1:**
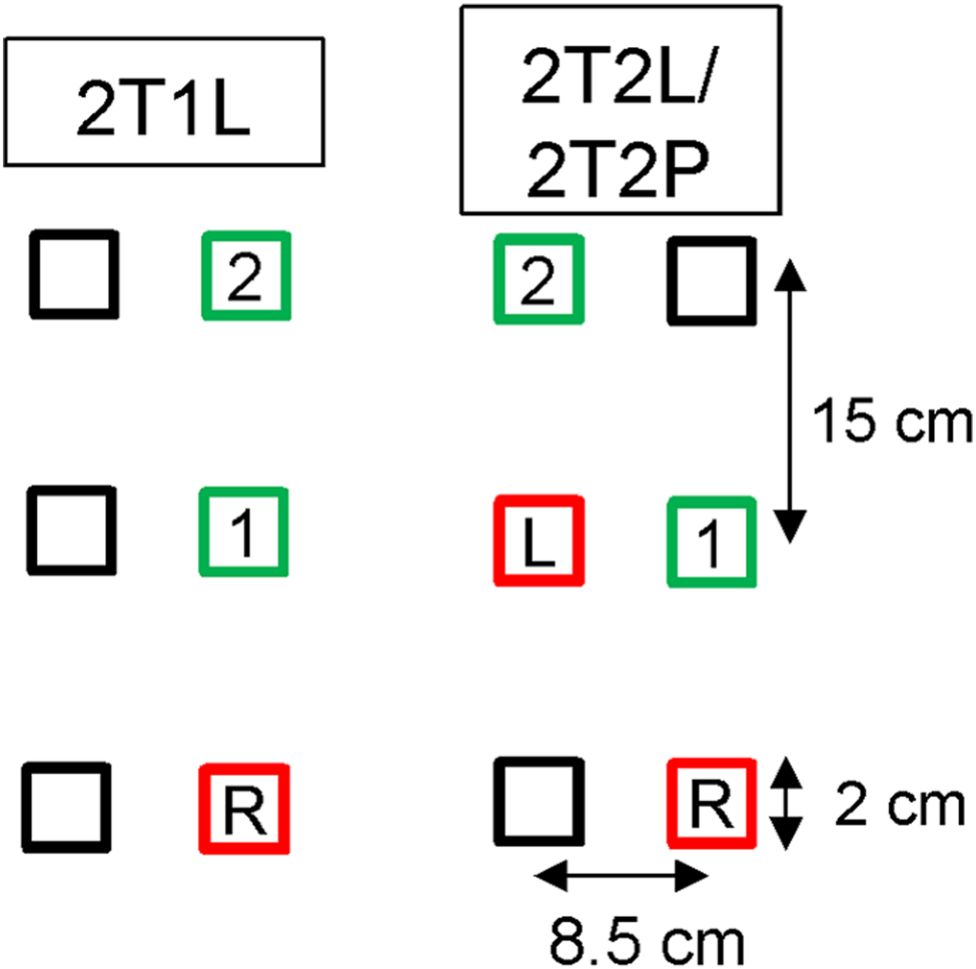
Illustration of the target array and sequence aiming contexts. Movements of the right and left limbs are indicated by the letters *R* and *L*, respectively. Start and aimed target locations are highlighted by red and green outlines, respectively. The order of movement segments is indicated by the numbers (1, 2)

Participants made a single visit to the lab in pairs. They assumed a sitting position in one of two chairs, which were positioned directly alongside each other. Here, they would execute aims with their upper limbs by pointing their index finger toward targets as quickly and accurately as possible. The target array was an illustration of two sets of two targets and a home position, which were each coloured in red (see Fig. 1). The targets and home position were 2-cm squares, and each separated by 15 cm (centre-to-centre) in the primary (sagittal) axis of the required movement and 8.5 cm in the secondary (frontal) axis (centre-to-centre). The right- and left-sided targets were designated for ipsilateral right- and left-handed aims, respectively.

Participants were instructed to execute a sequence of rapid aiming movements between two targets using only their right hand, both hands, or between pairs. The single-limb context simply involved the right limb aiming toward the first target followed by an immediate extension toward the second target (e.g., Adam et al. 2000) (two-target + one-limb; 2T1L). Meanwhile, the two-handed context initially involved having the left limb locate the start position at the first target with the right limb generating the first aim followed by the left limb generating the second aim (e.g., Khan et al. 2010) (two-target + two limbs; 2T2L). The final sequence aiming context involved pairs of participants sitting next to each other with the right-sided participant using their right limb to generate the first aim, and the left-sided participant using their left limb to generate the second aim (two-target + two-person; 2T2P).

For each context, participants were reminded to quickly and accurately execute the movements as a single sequence. Thus, while the first movement was initiated at a time of the participants’ own choosing (no external cue), the second movement would commence as quickly as possible after the first one was completed. For the single-person contexts (2T1L, 2T2L), the participant that was designated for aiming in that particular block would assume the seating position on the right side, while their partner vacated the seating position on the left side. While the pairs of participants did not sit directly alongside each other for these particular contexts, they were still clearly able to see each other including their aimed responses. There were 15 trials per sequence aiming context (total = 45 trials), which were ordered in a blocked fashion with blocks being counter-balanced between participants using a Latin-Square design. Pairs of participants took it in turns to complete each block of trials, which meant that they would simply switch roles for the two-person aiming context (i.e., leader → follower, follower → leader).

Movements were recorded using a Vicon camera system (Vicon Vantage, 16-megapixel resolution) sampling at 200 Hz, which detected retro-reflective markers that were attached to the left and right index fingers. Each trial was manually selected to commence recording for a period of 3 secs allowing the participants to completely execute the required aiming movement. Data collection and marker reconstruction were controlled via Vicon Nexus software.

### Data management and analysis

Cartesian coordinates were filtered using a dual-pass Butterworth filter to the order of 2 with a low-pass cut-off frequency of 10 Hz. Position data were differentiated via the three-point method to obtain velocity. Movements within each segment were identified by manually picking the first and second sets of velocity peaks within the vertical (z-)axis courtesy of a graphical user-interface within Matlab (R2018b) (The Mathworks Inc., Natick, MA). To indicate the moment of movement onset within each segment, data were parsed backward frame-by-frame from the maximal velocity peak until reaching < 20 mm/s^1^. Meanwhile, the moment of movement offset within each segment was indicated by parsing forward from the minimal velocity peak until reaching > −20 mm/s.

Dependent measures included overall movement time, time to and after peak velocity, displacement at peak velocity and movement end, and magnitude of peak velocity. The means for each of our measures of interest were analysed separately for the first and second segments. Additionally, we extracted the within-participant standard deviation of the movement time, as well as the spatial variability at kinematic landmarks (peak velocity, movement end). Notably, the measures that precede peak velocity are primarily attributed to the pre-response planning of movement, while the measures thereafter pertain to the online control in approach to the end target (e.g., Hansen et al. 2008).

Dependent measures from the first and second segments were first analysed using one-way repeated-measures Analysis of Variance (ANOVA), which consisted of three levels of context (2T1L, 2T2L, 2T2P). Meanwhile, spatial variability was analysed using a two-way ANOVA with repeated-measures factors of landmark (peak velocity, movement end) and context (2T1L, 2T2L, 2T2P). As a result of the previous finding that temporal variability coincides with the coordination or integration of movements between pairs (Vesper et al. 2011), we additionally correlated the within-participant temporal variability of the first segment with the participant mean pause times for the 2T2P context. What is more, if perception and action are directly coupled in the two-person context, it was reasonable to assume that there would be a positive relation between the observed events of the first segment and the executed events of the second segment (for a similar logic, see Khan et al. 2011; Roberts et al. 2016). Thus, we calculated Fisher z-transformations of the within-participant correlations between the first and second segments. Each score from the movement contexts was compared using one-way repeated-measures ANOVA, as well as a comparing with a theoretical value of zero courtesy of single-sample t tests. Indeed, it was reasoned that if perception and action are directly coupled in the two-person context, then the execution of the second segment should positively co-vary with the observed events of the first segment.

In the event of a violation of Sphericity (as assessed by Mauchly’s test of Sphericity), the Huynh–Feldt correction was adopted when epsilon was > 0.75 with the Greenhouse–Geisser value being adopted if otherwise (original Sphericity-assumed degrees of freedom were nonetheless reported). Effect sizes were indicated courtesy of partial eta-squared (*ƞ*^*2*^) and significant effects consisting of multiple means were decomposed using the Tukey HSD post hoc procedure. Significance was declared at *p* < 0.05.

## Results

### First movement segment

**Fig. 2:**
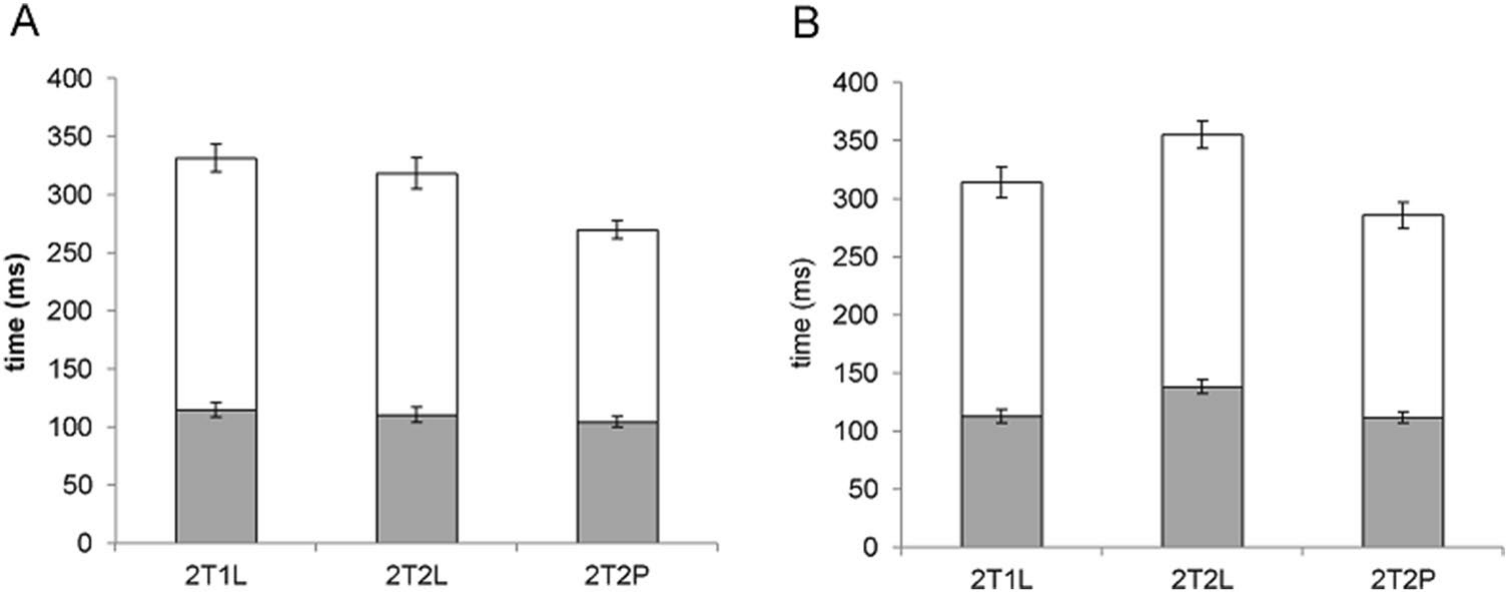
Mean movement times within the first (**a**) and second (**b**) segment as a function of sequence aiming context. Times are brokered into the time to (grey bars), and after (white bars), peak velocity. Error bars indicate standard error of the mean

There was a significant main effect of context for movement time, *F*(2, 46) = 11.30, *p* < 0.05, partial ƞ^2^ = 0.33, indicating a shorter time within movement for the 2T2P compared to 2T1L and 2T2L (*ps* < 0.05) (see Fig. 2a). This significant effect was not reflected in the time to peak velocity, *F*(2, 46) = 1.67, *p* > 0.05, partial ƞ^2^ = 0.07, but the time after peak velocity, *F*(2, 46) = 13.71, *p* < 0.05, partial ƞ^2^ = 0.37.

For the displacement at kinematic landmarks, there was a significant main effect at peak velocity, *F*(2, 46) = 9.69, *p* < 0.05, partial ƞ^2^ = 0.30, which indicated a greater distance travelled during the 2T2P context compared to both remaining contexts (see Table 1) (*ps* < 0.05). There was a significant main effect at the end of the movement, *F*(2, 46) = 4.75, *p* < 0.05, partial ƞ^2^ = 0.17, which also indicated a longer reach for the 2T2P compared to 2T2L (*p* < 0.05), whilst the 2T1L occupied a non-significant intermediate level of endpoint displacement (*ps* > 0.05)^2^. Moreover, the magnitude of peak velocity revealed another significant main effect, *F*(2, 46) = 32.64, *p* < 0.05, partial ƞ^2^ = 0.59, which indicated a significantly larger impulse for the 2T2P compared to 2T1L and 2T2L (*ps* < 0.05).

**Table 1:** Means (± SE) of kinematic dependent measures within segment 1 and segment 2 as a function of sequence aiming context

		2T1L	2T2L	2T2P
Segment 1	Displacement at peak velocity (mm)	60.45 (1.91)	63.86 (1.81)	69.56 (0.98)
	Displacement at movement end (mm)^a^	149.57 (1.00)	148.85 (0.96)	151.88 (0.77)
	Magnitude of peak velocity (mm/s)	824 (39)	903 (48)	1123 (45)
Segment 2	Displacement at peak velocity (mm)	61.29 (2.48)	66.74 (1.47)	68.97 (1.45)
	Displacement at movement end (mm)^b^	143.21 (1.65)	150.38 (.94)	150.79 (.84)
	Magnitude of peak velocity (mm/s)	798 (34)	780 (31)	1083 (.52)

^a^Segment 1 target error rate (%; as defined by reaching under (< 140 mm) or over (> 160 mm) the target) indicated no significant main effect of context, *F*(2, 46) < 1, *partial *ƞ^2^ = 0.04

^b^Segment 2 target error rate indicated a significant main effect of context, *F*(2, 46) = 6.24, *p* < 0.05, *partial ƞ*^2^ =0 .21, which indicated a larger proportion of errors for the 2T1L compared to 2T2L and 2T2P contexts (*ps* < 0.05). Crucially, there was no significant difference between the 2T2L and 2T2P contexts (*p* > 0.05) because the start position of the second segment was always near a set location (i.e., centre of the first target)

For spatial variability, there was a significant main effect of landmark, *F*(1, 23) = 12.95, *p* < 0.05*, *partial ƞ^2^ = 0.36, although no significant main effect of context, *F*(2, 46) < 1, partial ƞ^2^ = 0.01. However, these effects were superseded by a significant landmark x context interaction, *F*(2, 46) = 5.34, *p* < 0.05, partial ƞ^2^ = 0.19. Simple effect analyses confirmed a significant effect at peak velocity, *F*(2, 46) = 12.14, *p* < 0.05, partial ƞ^2^ = 0.14, although no significant effect at movement end, *F*(2, 46) < 1, partial ƞ^2^ = 0.08. Post hoc analysis revealed that there was significantly smaller variability for the 2T2P context compared to 2T2L (*p* < 0.05), and a similar but non-significant trend for the comparison with 2T1L (*p* < 0.1) (see Table 2)^3^. Finally, the correlation between the within-participant temporal variability at the first segment (*M* = 37.78 ms, SE = 3.73) and the participant mean pause time between segments (*M* = 10.10 ms, SE = 12.27) for the 2T2P context revealed a significant positive relation, *r* = 0.50, *p* < 0.05^4^

**Table 2:** Mean (± SE) spatial variability at kinematic landmarks within segment 1 and segment 2 as a function of sequence aiming context

		2T1L	2T2L	2T2P
Segment 1	Variability at peak velocity (mm)	8.43 (0.91)	8.86 (1.13)	7.00 (0.64)
	Variability at movement end (mm)	5.01 (0.47)	5.13 (.33)	6.27 (0.63)
Segment 2	Variability at peak velocity (mm)	9.62 (0.95)	11.00 (1.36)	8.86 (0.66)
	Variability at movement end (mm)	8.01 (1.27)	6.82 (0.99)	7.22 (0.73)

### Second movement segment

There was a significant main effect of context for movement time, *F*(2, 46) = 12.08, *p* < 0.05, partial ƞ^2^ = 0.34, which indicated a significantly longer time to completion for the 2T2L context compared to 2T1L and 2T2P (*ps* < 0.05), which were not significantly different from each other (*p* > 0.05) (see Fig. 2b). This pattern of results was primarily reflected by the significant effect for time to peak velocity, *F*(2, 46) = 8.35, *p* < 0.05, partial ƞ^2^ = 0.27. However, the significant effect for time after peak velocity, *F*(2, 46) = 7.05, *p* < 0.05, partial ƞ^2^ = 0.24, revealed a significantly shorter time for the 2T2P compared to 2T2L (*p* < 0.05), and a similar trend for the comparison with 2T1L (*p* < 0.1).

For displacement at kinematic landmarks, there was a significant main effect at peak velocity, *F*(2, 46) = 4.54, *p* < 0.05, partial ƞ^2^ = 0.17, which indicated a longer initial reach for the 2T2P context compared to 2T1L (*p* < 0.05) (see Table 1). Additionally, there was a significant main effect at movement end, *F*(2, 46) = 15.03, *p* < 0.05, partial ƞ^2^ = 0.40, as the 2T1L context was significantly shorter than both remaining contexts (*ps* < 0.05), which failed to significantly differ from each other (*p* > 0.05). For the magnitude of peak velocity, there was a significant main effect, *F*(2, 46) = 15.03, *p* < 0.05, partial ƞ^2^ = 0.40, indicating a significantly higher impulse for the 2T2P context compared to each of the remaining contexts (*ps* < 0.05).

With regard to spatial variability, there was a significant main effect of kinematic landmark, *F*(1, 23) = 21.96, *p* < 0.05, partial ƞ^2^ = 0.49, although no significant main effect of context, *F*(2, 46)  <  1, partial ƞ^2^  =  0.01, nor a significant landmark × context interaction, *F*(2, 46) = 2.29, *p* > 0.05, partial ƞ^2^ = 0.09 (see Table 2).

Finally, for the within-participant correlations, we primarily isolated our analyses to the measures that indicated a similar effect of context within the first and second segments^5^. There were no significant differences in the correlations formed for the time after peak velocity, *F*(2, 42) = 2.13, *p* > 0.05, partial ƞ^2^ = 0.09, displacement at peak velocity, *F*(2, 42) < 1, partial ƞ^2^ = 0.01, and magnitude of peak velocity, *F*(2, 42) = 1.88, *p* > 0.05, partial ƞ^2^ = 0.08. Nevertheless, there was a significant positive relation between the first and second segment times after peak velocity for 2T1L (*M* = 0.39, SE = 0.09), *t*(21) = 4.50, *p* < 0.05, and 2T2P (*M* = 0.27, SE = 0.12), *t*(21) = 2.27, *p* < 0.05, although the significant relations identified for the magnitude of peak velocity were restricted to only the 2T1L context (*M* = 0.29, SE = 0.10), *t*(21) = 2.81, *p* < 0.05. Meanwhile, there were no significant relations between the first and second segment displacements at peak velocity for any of the contexts (*ts* < 1; grand* M* = 0.02, SE = 0.09).

## Discussion

The present study simultaneously aimed to examine a priori task co-representation and continuous perception–action coupling within a single task context. Participants executed either sequence aiming movements individually or in pairs by having a second person continue the sequence that was initially undertaken by the first person. By definition, we demarcated the roles of each person so one was designated as the leader, and the other as follower, respectively. That is, the effects found for the leader could be attributed to the sharing of anticipated constraints within the follower’s movement, while the follower could additionally rely on the spatio-temporal characteristics of the observed movement from the leader. Thus, we predicted that the leader would accommodate the follower by enhancing their speed, as well as restricting their temporal variability. Meanwhile, the follower could closely replicate the spatio-temporal kinematics that were previously generated by the leader. The following discussion will systematically explore the findings for each of these areas.

### Leader effects

The initial movement from the designated leader was executed more quickly, which was primarily designated to the time after peak velocity. While this finding would normally indicate more rapid online control, where error corrections are made based on feedback from within the movement (Elliott et al. 2001; Khan et al. 2003), it is perhaps more likely that performers relied more upon pre-response planning without greatly updating the movement. This interpretation coincides with reduced spatial variability (for similar findings within a reaching and grasping task, see Sacheli et al. 2013) despite there being a higher magnitude of peak velocity (see Meyer et al. 1988; Schmidt et al. 1979). Thus, it is possible that performers altered their pre-response planning by keeping the spatial variability of the initial movement comparatively low to minimise the need for online control later within the movement (Allsop et al. 2017; Roberts et al. 2018), while limiting the negative effects on endpoint accuracy and precision (Khan et al. 2002; see also, Fischman and Reeve 1992). Indeed, pre-response planning usually entails the optimal selection or parameterization of movement that most likely limits the inherent sources of variability (Hamilton and Wolpert 2002; Hamilton et al. 2004; Harris and Wolpert 1998; see also, van Beers 2009).

Consequently, it would appear that the two-person context featured a generally greater feedforward approach, which involved less online control following a more precise parameterization of the movement. On the other hand, the single-person contexts (2T1L, 2T2L) featured a comparatively feedback-based approach, where performers could more greatly anticipate the need to make a correction following a perceived error within the movement. That said, the underlying reason behind these different approaches depending on whether the movements were made in a joint or individual action context remains to be seen. For example, while participants were able to equally observe and be present for their partner’s responses across each of the different contexts, it is possible that the more interactive and communicative nature of the present joint action context may have additionally involved an inherent drive (i.e., phylogenetic) toward a social exchange (Csibra and Gergely 2011).

Meanwhile, there was evidence of a significant relation between the temporal variability of the leader and the time spent initiating the second movement from the follower. Thus, a smaller variation in the time to complete the first movement from the leader coincided with a more rapid initiation of the second movement from the follower. These findings correspond with those of Vesper et al. (2011), which indicated that reaction time variability was decreased to accommodate synchrony with another co-actor executing rapid limb movements. That is, the decrease in variability makes the movement more predictable for the co-actor, and thus easier for them to respond to. In this regard, the leader may adapt their movement in such a way that it helps communicate with or signal the follower to execute a complementary rapid response (Sacheli et al. 2013; Vesper et al. 2010; see also Wolpert et al. 2003). Such preparatory processes on behalf of the leader may involve the same neural network that is often associated with the direct observation and execution of actions within real-time (Cavallo et al. 2013; Kilner et al. 2004; Ramnani and Miall 2004).

### Follower effects

Comparatively speaking, the overall movement time effects from the follower seemed to contrast with those from the initial leader. Specifically, there was an equally shorter movement time for the one-limb and two-person contexts compared to the two-limb context, which was solely attributed to the time to peak velocity. Presumably, the slower within-person response when transitioning between two limbs manifested from some independent cost of a bimanual or inter-hemispheric transfer (i.e., right-to-left limb movement; e.g., Heath et al. 2007).

However, closer assessment of the other relevant measures appeared to indicate some replication of the leader’s movement. That is, there was a similarly shorter time after peak velocity, as well as a longer displacement and magnitude of peak velocity for the two-person context compared to both the single-person contexts. It could be argued that this similar pattern of results was an artefact of social facilitation (Zajonc 1965), where both performers inadvertently reduced the time spent ‘homing-in’ because they were merely witnessing each other’s responses. However, this explanation remains doubtful given there were some opposing effects between each of the roles including reduced spatial variability within the leader, but not so for the follower.

To corroborate the effects from the follower, our within-participant correlations confirmed that there was a significant relation between the times spent after peak velocity in the first and second segments. Consistent with this finding is evidence from imitation learning in which observers of novel digitized movement kinematics tend to correspondingly re-distribute their typical velocity–time profile (i.e., exaggerated asymmetry in the relative time after peak velocity; Hayes et al. 2014, 2016). Based on these findings, it appears that the follower observed, and potentially embodied, the movement of the leader by replicating the temporal parameters of their impulse and control phases.

### Combined joint action effects

Taken together, these findings advocate theoretical frameworks that infer two distinct levels of joint action control: top–down co-representation of task constraints and bottom–up simulation of observed action events. Indeed, a growing number of models, including predictive coding (Kilner et al. 2007) and minimal architecture (Vesper et al. 2010), may be coarsely categorised as such. By analogy, we can draw upon the research findings indicating different levels of interference, including the conceptual- and movement-related (e.g., Ondobaka et al. 2012; Roberts et al. 2017). These particular findings reflect how the correspondence between the intentions of observed and executed actions dictate the extent of lower-level movement interference. That is, the tendency to have ones movements (e.g., move to the leftward item) slowed by incongruent movement observation (e.g., move to the rightward item) can be enhanced providing there is some relation between the observer and co-actor’s action intentions (e.g., select the same type of item). In the context of the present findings, it appears that the anticipatory and accommodative measures taken by the leader are synonymous with the proposed conceptual level, whilst the tendency for the follower to copy observed movements reflects the mirroring of observed into executed movements.

In conclusion, the present study may uniquely pose a single task paradigm that simultaneously ascertains the multiple levels of operation for joint action. Indeed, we adopted a covert joint action context, where performers seek to move to the same target goal by integrating the entire sequence of movements between pairs (leader) or continuing to extend upon the movement previously observed (follower). As a result, the current study contributes to a growing trend in joint action research to incorporate multi-segment sequences (e.g., Schmitz et al. 2018) that more closely comprise continuous measures of performance (e.g., spatio-temporal characteristics; Rocca and Cavallo 2020).

## Data Availability

The datasets generated during and/or analysed during the current study are available from the corresponding author on reasonable request.

## Footnotes

1 Following initial attempts to adopt the primary movement axis for indicators of the start and end of individual segments, there were some potential instances of sliding or a continually high velocity magnitude when transitioning between segments. Thus, we adopted a previously evidenced criterion for motion capture within sequence aiming (Khan et al. 2011)

2 The nature of the extended displacements at peak velocity and movement end for the 2T2P context (Table 1) may suggest that they were not necessarily attributed to the same process. Indeed, it could be argued that the extended displacement at movement end was a mere remnant of the initially extended displacement at peak velocity and reduced time after peak velocity, where there was less space and time devoted to decelerating the limb and overcoming the existing inertia. In line with this logic, an analysis of the distance travelled between peak velocity and movement end indicated a significant main effect of context, *F*(2, 46) = 6.02, *p* < 0.05, *partial ƞ*^2^ = 0.21, which indicated a shorter distance travelled by the 2T2P compared to the 2T1L (*p* < 0.05). There were no other significant pairwise differences (*ps* > 0.05). Thus, there appeared a genuine attempt to equalise the displacements across the different contexts as the limb neared the end of the movement.

3 In light of the variability findings, it is somewhat surprising granted the inversely larger magnitude of velocity that was generated by the 2T2P context. To corroborate our findings, we additionally assessed the coefficient of variation (spatial variability/mean displacement) as refined parameterization and/or advanced online control would be indicated by a comparatively low value. This reasoning is adapted from the notion that variability exponentially increases with amplitude (see Khan et al. 2006) meaning any sudden decreases in variability relative to amplitude must indicate some adaptive sensorimotor process. Using the same statistical design as the spatial variability measure, the analysis confirmed another significant landmark x context interaction, *F*(2, 46) = 8.52, *p* < 0.05, partial ƞ^2^ = 0.27, with a lower score at peak velocity for the 2T2P context (*M* = 0.13, SE = 0.01) compared to 2T1L (*M* = 0.17, SE = 0.03) and 2T2L (*M* = 0.17, SE = 0.02) (*ps* < 0.05). The same analysis conducted at the second segment failed to reveal a significant landmark x context interaction, *F*(2, 46) = 1.48, *p* > 0.05, partial ƞ^2^ = 0.06 (grand* M* = 0.10, SE = 0.02)..

4 An analysis of the mean pause times showed a significant main effect of context, *F*(2,46) = 15.97, *p* < 0.05, partial ƞ^2^ = 0.41. Post hoc analysis indicated a significantly larger pause time for the 2T1L (*M* = 80.36 ms, SE = 18.05) compared to 2T2L (*M* = −7.49 ms, SE = 15.04) and 2T2P (*M* = 7.63 ms, SE = 14.16) (*ps* < 0.05). Meanwhile, there was no significant difference between the 2T2L and 2T2P (*p* > 0.05). On review of the mean pause times, the 2T1L context was sufficiently short that it appeared the two segments were pre-planned in advance and near fully integrated (Khan et al. 2010; Lawrence et al. 2016), while there was a degree of overlap between the first and second segments for the 2T2L and 2T2P contexts.

5 A further two participants were removed (*n* = 22) from the analysis of the within-participant correlations as their individual trial data could not be correlated with their partners who were previously removed due to data recording errors (see *Participants* sub-section).
